# Influence of Spatial and Chromatic Noise on Luminance Discrimination

**DOI:** 10.1038/s41598-017-16817-0

**Published:** 2017-12-05

**Authors:** Leticia Miquilini, Natalie A. Walker, Erika A. Odigie, Diego Leite Guimarães, Railson Cruz Salomão, Eliza Maria Costa Brito Lacerda, Maria Izabel Tentes Cortes, Luiz Carlos de Lima Silveira, Malinda E. C. Fitzgerald, Dora Fix Ventura, Givago Silva Souza

**Affiliations:** 1 Instituto de Ciências Biológicas, Universidade Federal do Pará, Belém, Pará Brazil; 2 Núcleo de Medicina Tropical, Universidade Federal do Pará, Belém, Pará Brazil; 3 University of Tennessee Health Science Center, Memphis, TN United States of America; 4 Christian Brother’s University, Memphis, TN United States of America; 5 Universidade Ceuma, São Luiz, Maranhão Brazil; 6 Instituto de Psicologia, Universidade de São Paulo, São Paulo, São Paulo Brazil; 7 Universidade Federal do Amapá, Macapá, Amapá Brazil

## Abstract

Pseudoisochromatic figures are designed to base discrimination of a chromatic target from a background solely on the chromatic differences. This is accomplished by the introduction of luminance and spatial noise thereby eliminating these two dimensions as cues. The inverse rationale could also be applied to luminance discrimination, if spatial and chromatic noise are used to mask those cues. In this current study estimate of luminance contrast thresholds were conducted using a novel stimulus, based on the use of chromatic and spatial noise to mask the use of these cues in a luminance discrimination task. This was accomplished by presenting stimuli composed of a mosaic of circles colored randomly. A Landolt-C target differed from the background only by the luminance. The luminance contrast thresholds were estimated for different chromatic noise saturation conditions and compared to luminance contrast thresholds estimated using the same target in a non-mosaic stimulus. Moreover, the influence of the chromatic content in the noise on the luminance contrast threshold was also investigated. Luminance contrast threshold was dependent on the chromaticity noise strength. It was 10-fold higher than thresholds estimated from non-mosaic stimulus, but they were independent of colour space location in which the noise was modulated. The present study introduces a new method to investigate luminance vision intended for both basic science and clinical applications.

## Introduction

The natural environment is composed of a mosaic of adjacent patches that reflects different numbers of photons with distinct spectral content, which is the substrate that the visual system builds on for the perceptual experience of colour and luminance^1^. Colour and luminance information may have had a major role in the evolution of the visual system^2^ that might explain why different cells have evolved to encode colour and luminance differences in both temporal and spatial domains^3^. The colour and luminance contrast of an image modulates the activity of ganglion cells within the retina^3,4^. At least three groups of ganglion cells encode chromatic isoluminant contrast and luminance contrast with different contrast sensitivities^4–6^. M cells have high luminance contrast sensitivity and weak responses for chromatic isoluminant contrast^4,5,7–14^. P cells have high contrast sensitivity for red-green isoluminant contrast and low luminance contrast sensitivity^4,5,7–13^. Small bistratified ganglion cells (K cells) are highly responsive to blue-yellow isoluminant contrast and have little or no responsiveness to luminance contrast^5,15^. The signals of luminance and colour contrast are transmitted from the retina to the lateral geniculate nucleus (LGN) and from there to the primary visual cortex (V1) through parallel streams named M, P, and K, respectively^16–18^.

In V1, the cortical cells receive inputs from M, P, and K-pathways, although it is not clear the weighting of the input to the individual neurons^19–21^. It is also not clear if the luminance and colour contrast interacts to modulate the neuronal response prior to the synaptic connection within V1^5^; however, evidence does suggest that it occurs within V1^22–24^. Two different studies have^22,23^ reported three groups of V1 cells in the macaque with preferences for luminance contrast, for colour contrast, and for both luminance and colour contrasts.

Additional studies have reported results on how the visual system detects a stimulus with simultaneous luminance and colour contrasts^23,25–29^. These results were that colour and luminance channels are not entirely independent, and each can exert an impact in the functionality of the other^23,25–29^.

Pseudoisochromatic stimuli are composed of a combination of luminance and colour contrast^30^. The design of the stimuli is a mosaic that has a spatial noise added to a luminance noise within its patches. The target highlights from the mosaic field by the chromaticity difference^31^. Pseudoisochromatic stimulus is widely used to investigate colour discrimination. In this particular type of test, the chromatic contrast is embedded in luminance and spatial noise, and the subject performs a colour discrimination task^28,29,31–36^. Other stimulus design have been used to investigate the colour discrimination^37,38^.

Our research group has described that different luminance and colour interactions within the pseudoisochromatic stimuli can have an influence on colour discrimination tasks^28^. We investigated how the number of luminance values in the luminance noise influenced the colour perception^28^. We observed that when the stimuli have two or four luminance values in the luminance noise the color discrimination was worse than when the stimuli had six or more luminance values in the luminance noise. We also observed that the colour discrimination thresholds and reaction times were dependent on the relationship between the mean luminance of the mosaic and the range of the luminance noise^29^.

In the present investigation, a novel stimulus was introduced that could be used to investigate the influence of the luminance and color interactions within the luminance discrimination tasks. This new stimulus, similar to the pseudoisochromatic stimulus, is also composed of a mosaic with spatial noise, but it has chromatic noise randomly distributed within the mosaic. The chromatic noise, along with spatial noise, mask the presence of a target that differs from the mosaic field by luminance contrast. We investigated the influence of this chromatic noise on the luminance discrimination thresholds as well as the influence of the mosaic design in the measurement.

## Results

### Experiment #1. Luminance contrast threshold as a function of the chromatic noise length

**Figure 1:**
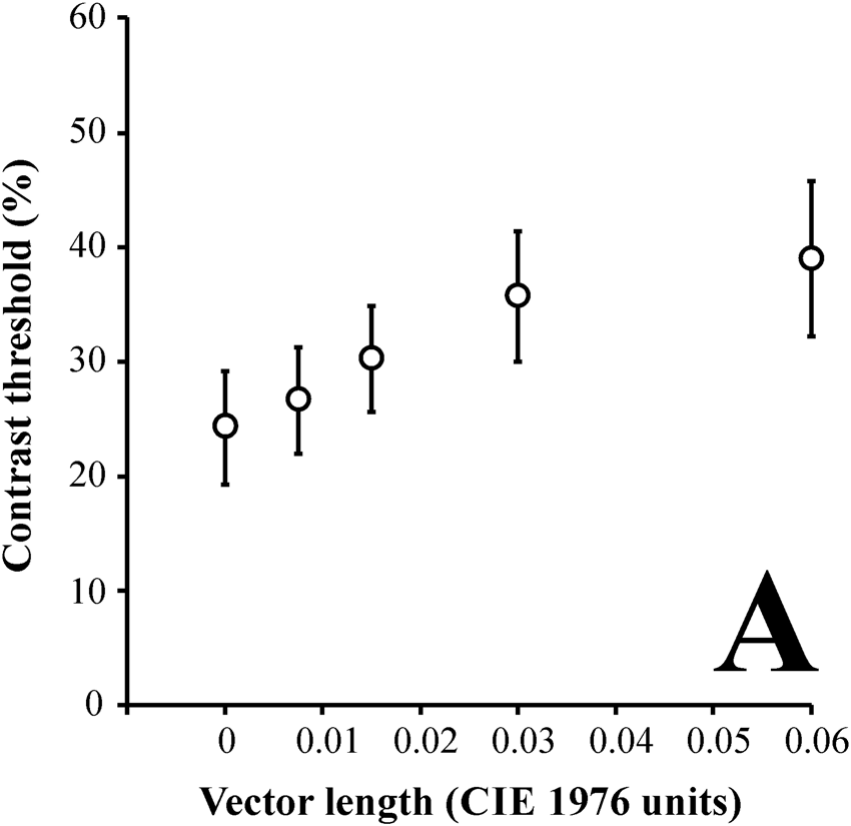
Mean luminance contrast thresholds (n = 40) as a function of chromatic noise length. The higher magnitude of noise, the higher luminance contrast threshold. Error bars represent the standard deviation of the mean.

The luminance contrast thresholds varied as a function of the vector length of the chromatic noise values. The presence of the chromatic noise was associated with a reduction in the luminance discrimination threshold. The higher chromatic noise length, the higher luminance contrast threshold. Figure 1 shows the mean contrast threshold (n = 40, 2 trials) as a function of the chromatic noise (Fig. 1). The mean luminance thresholds for the different chromatic noise conditions (in u’v’ units) were: 0.06: 38.9% ± 6.8; 0.03: 35.6% ± 5.7; 0.015: 30.2% ± 4.6; 0.0075: 26.6% ± 4.6; and 0: 24.2% ± 5.

**Figure 2:**
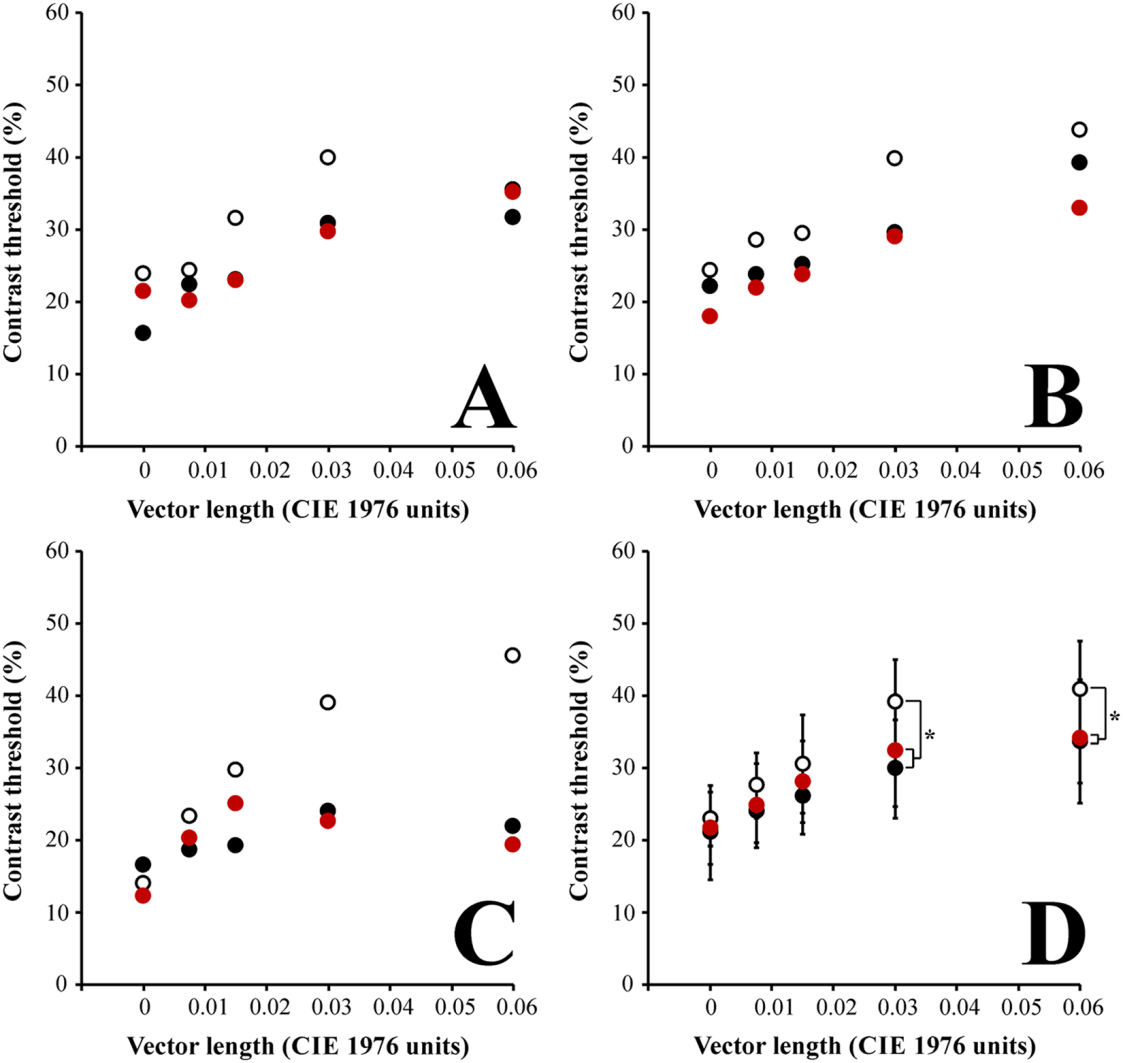
Comparison of luminance contrast thresholds as a function of the chromatic noise length obtained in three consecutive sessions (white circles, first session; black circles, second session; red circles, third session). (**A**–**C**) represent individual datapoints from three representative participants of the sample. (**D**) represented the averaged functions for each session. There were significant differences for the chromatic noise vectors of 0.06 and 0.03. The first session had higher contrast thresholds than the other two consecutive sessions (*p < 0.05). Error bars represent the standard deviation of the mean.

For a subgroup of observers, we compared the luminance contrast thresholds in three consecutive sessions. Figure 2 shows the comparison of three individual results (Fig. 2A–C) and group results of the sessions 1, 2, and 3. We observed that all the sessions showed a inhibitory influence of the chromatic vector length in the luminance contrast thresholds. We found statistical difference between the luminance contrast threshold obtained with chromatic noise of 0.06 and 0.03 u’v’ units. The contrast thresholds estimated for these stimuli in the first session were significantly higher than those obtained for the same conditions during the second and third session (Fig. 2D, p < 0.05). All the other intersession comparisons for the same chromatic noise length had no significant differences.

### Experiment #2. Comparison of luminance contrast threshold estimated using mosaic and non-mosaic stimulus

**Figure 3:**
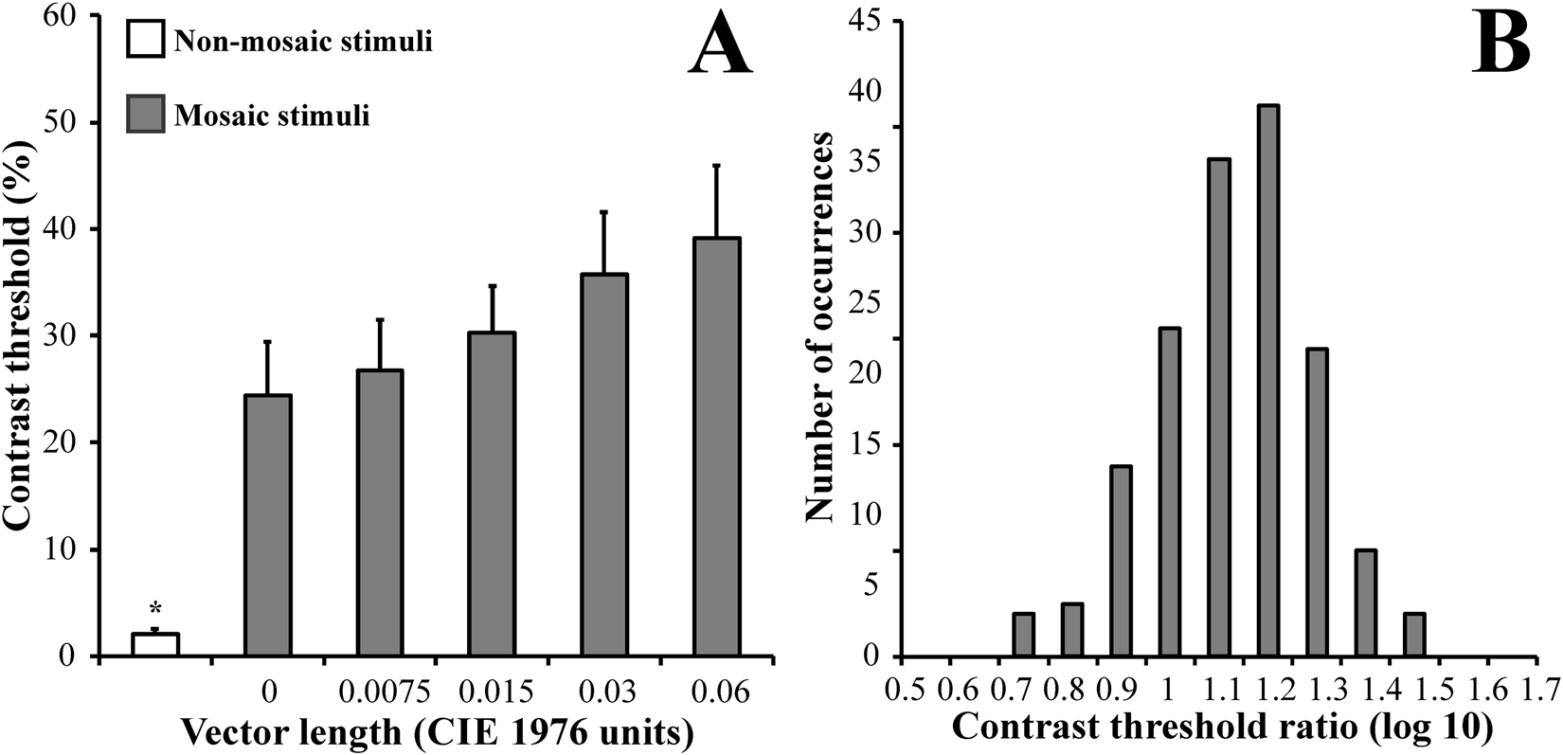
Comparison of the luminance contrast thresholds obtained using mosaic (gray bars) and non-mosaic (white bar) stimuli (**A**). The thresholds estimated using mosaic stimulus were significantly higher than those estimated using non mosaic stimulus for all subjects at all conditions. (**B**) Normal frequency distribution of the log10 ratio between the thresholds estimated using mosaic stimulus and non-mosaic stimulus. The median of the distribution was 1.18 log units. Error bars represents the standard deviation (n = 40). Error bars represent the standard deviation of the mean.

Figure 3 illustrates the comparison of luminance contrast thresholds estimated using mosaic stimulus at the different chromatic noise conditions (the same from the Experiment #1) and those estimated using non-mosaic stimulus. All contrast thresholds obtained with the mosaic stimulus were significantly higher than that estimated with the non-mosaic stimulus (p < 0.01). The frequency distribution of the log10 of the ratio between the thresholds estimated with mosaics and the threshold estimated using non-mosaic stimulus are shown in Fig. 3B. The data had a normal distribution and the median of the log10 of the ratio of the distribution was 1.19, the first quartile was 1.08, and the third quartile was 1.28.

### Experiment #3. Comparison of the luminance contrast thresholds estimated using stimuli with different chromatic noise content

**Figure 4:**
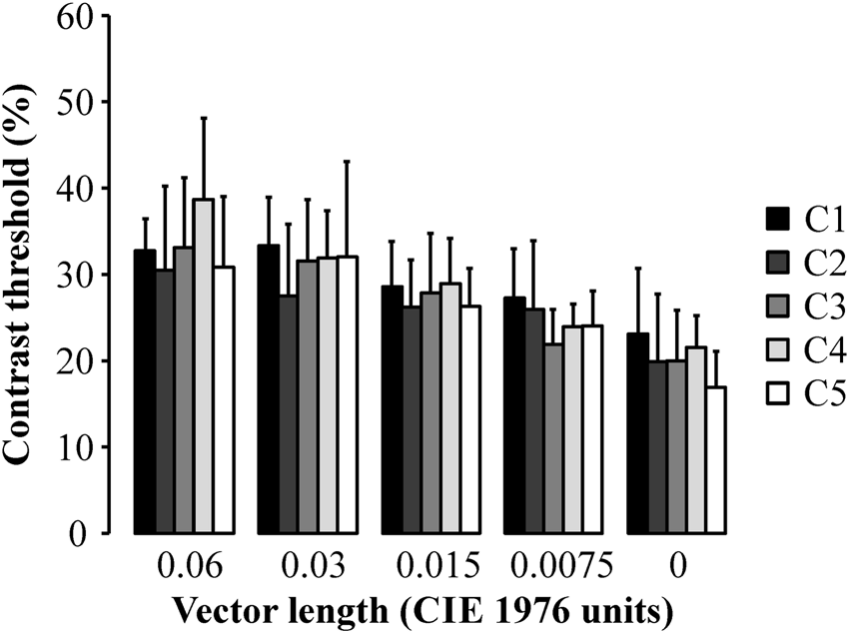
Comparison of the contrast thresholds estimated using five mosaics with different chromatic content (n = 7). There was no significant difference between the thresholds estimated using each one of the adapting chromatic state for the same chromatic noise length. Error bars represent the standard deviation of the mean.

Seven observers carried out the experiment #3. No difference was observed in the contrast thresholds estimated from a same chromatic noise length around the five reference chromaticities (p > 0.05). Figure 4 shows the multiple comparisons of contrast threshold obtained from five different chromatic noises.

### Comparison of the luminance contrast thresholds estimated using liquid crystal display (LCD) and cathode ray tube (CRT)

**Figure 5:**
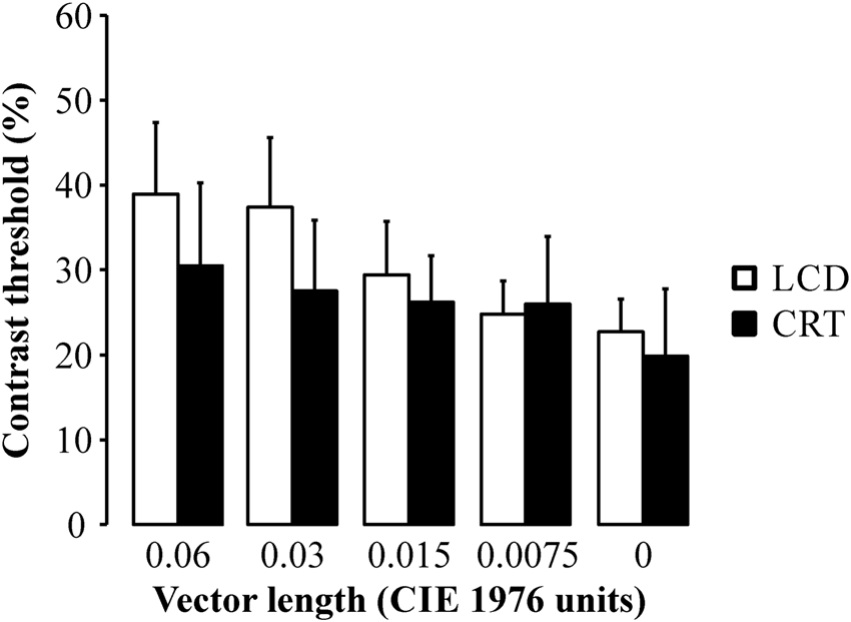
Comparison of the luminance contrast thresholds (n = 7) estimated from stimulus presented in the LCD (white bars) and CRT (black bars) and chromatic noise generated around C2. There was no significant difference between the thresholds estimated from the same chromatic noise length. Error bars represent the standard deviation of the mean.

We compared the results obtained from the same observers (n = 7) tested using both screens and the chromatic noise generated from the C2 reference chromaticity (Fig. 5). There was no influence of the system used to stimulate the psychophysical experiment. For the same chromatic noise length, the luminance contrast thresholds estimated using LCD and CRT had no significant difference.

We also compared the results from these observers (n = 7) tested using CRT and LCD and subgroups of the sample tested using only LCD. To compose these subgroups tested using LCD, we randomly select 7 observers from 33 observers. The results of the multiple comparisons between CRT observers and LCD observers is shown in the Table 1.

**Table 1:** p-value of the analysis of variance comparing the luminance contrast threshold obtained using stimuli with different chromatic noise length presented in LCD (10 randomly chosen subgroups with 7 observers) and CRT (7 observers).

Condition (u’v’ units)	Comparison									
	#1	#2	#3	#4	#5	#6	#7	#8	#9	#10
*0.06*	<0.01	ns	<0.05	<0.05	ns	ns	<0.01	ns	ns	ns
*0.03*	<0.01	<0.05	<0.05	Ns	ns	ns	<0.01	ns	ns	ns
*0.015*	ns	ns	ns	Ns	ns	ns	<0.01	ns	ns	ns
*0.0075*	ns	ns	ns	Ns	ns	ns	ns	ns	ns	ns
*0*	ns	ns	ns	Ns	ns	ns	ns	ns	ns	ns

ns: no statistical significance.

We observed that after 10 comparisons between the CRT observer group and the LCD observer subgroups, 18% of the comparisons had statistical significance and it was quite constrained to the higher chromatic noise lengths.

## Discussion

A new mosaic stimulus was used to evaluate luminance discrimination in this current study. The luminance contrast was masked by chromatic and spatial noise randomly distributed in the mosaic. The most important observation was that the presence of chromatic noise impaired luminance discrimination. This was similar to what was observed using pseudoisochromatic stimulus^28,29^; therefore, this new mosaic stimulus can be used to investigate colour and luminance interactions on visual perception.

Most of the psychophysical studies that have combined colour and luminance in the stimulus have shown that colour influenced the luminance perception and vice-versa. This influence of chromatic information within the luminance discrimination is controversial. Some investigators have shown that interaction between colour and luminance increased the luminance contrast sensitivity^25,39^. These investigators observed that when color and luminance contrasts were combined, the contrast sensitivity was enhanced for several spatial frequencies, thus reflecting a facilitatory interaction between the chromatic and luminance systems^25,39^. An additional group investigated the effect of luminance masking in the chromatic discrimination and the effect of chromatic masking in the luminance discrimination^26^. It was reported that luminance masking improved the chromatic discrimination at all ranges of luminance contrast of the masking, while chromatic contrast masking in the luminance contrast stimulus had a double effect in the luminance discrimination^26^. Low chromatic contrast masking had no influence in the luminance discrimination, intermediate chromatic contrast masking impaired the luminance discrimination, and high chromatic contrast masking increased the luminance discrimination^26^. The results reported in the present investigation as well as previous studies^28,29^ confirmed previous results concerning masking experiments on visual perception. In that similar mechanisms govern the detection of luminance contrast in the presence of colour contrast, and detection of colour contrast in the presence of luminance contrast^40^. No significant subthreshold cross modal effect has been observed, but there was facilitation for the detection of one attribute in the presence of the other in a broad range of suprathreshold contrast. At high contrast, both colour and luminance inhibited the detection of the other contrast modality^40^.

Several models of how luminance and colour channels interact have been proposed. The existence of one pathway that shares the transduction of luminance and colour has not been supported by published data from several investigators^40–43^. It is well known that P, M, and K ganglion cells have responses for colour and luminance, but with different gains^5,11–13,15^, supporting the existence of multiples channels of luminance and colour from the retina to V1. After V1, distinct pathways for colour and luminance information could then combine the M, P, and K inputs with different weights in higher order neurons^44^.

The results from Experiment #1 showed an inhibitory effect of the presence of chromatic information on the luminance contrast discrimination. The responsible mechanism for the task in Experiment #1 had low contrast sensitivity. This was even after the exclusion of all chromatic noise from the stimulus, and all the circles of the mosaic field had the same chromaticity. In this experimental procedure, the luminance thresholds were quite high compared to the peak of the contrast sensitivity function normally observed in a healthy subject. Another clue to the potential mechanism involved in the luminance contrast discrimination masked by chromatic noise can be inferred from the results in Experiment #2. This protocol sought to determine if the presence of the mosaic would potentially explain the results of high contrast thresholds. When the same task was used to identify the C-gap orientation, but in a stimulus without the mosaic array, the response results returned lower contrast thresholds. These results corresponded to data reported previously under similar testing conditions^45,46^. The comparison of chromatic discrimination estimated using pseudoisochromatic stimulus with that used an array of four homogeneous squares, one of which had a different chromaticity showed impaired discrimination in the tasks using mosaic stimulus^47^. These investigators suggested that the observer needed different strategies to identify the chromatic differences in both kinds of stimuli^47^. In the display array of squares, a simple search strategy was used, while a scan strategy was used as the choice in order to determine the C gap orientation in pseudoisochromatic design. The mosaic structure would contribute to a loss of information within the target. In the current investigation it was found that the ratio between the contrast thresholds estimated using non-mosaic and mosaic stimulus was around 1 log unit. Many authors have reported 1 log unit as the ratio of the luminance contrast sensitivity between M and P pathways activity at the retina^48,49^, LGN^50–52^, and psychophysics^53^. The presence of the mosaic caused the main effect on the luminance contrast thresholds. It impaired the contrast detection compared to the non-mosaic condition. The mosaic design favored the activity of a low contrast discrimination mechanism, such as determined by the P pathway. During our experiments, we used 16 different spatial noises during the experiments, while Cambridge Colour Test 2.3 version keeps the spatial noise constant during the test. There was no systematic investigation to evaluate the specific effects of the random or constant spatial noise in the luminance or colour discrimination. Future investigations can be done focused this question. Previous study investigated the chromatic discrimination thresholds with random spatial noise with and without luminance temporal noise and they found no differences on the thresholds obtained for normal trichromats^54^.

Added to this mosaic effect, we also found the influence of the chromatic noise on the luminance thresholds. Even in the chromatic noise condition of 0.0075 u’v’ units, we found impairment of the luminance contrast thresholds. The vector size of 0.0075 u’v’ units is just above to the colour discrimination thresholds observed for the subjects between 20 and 30 years^35^. This would indicate the existence of a mechanism with high chromatic discrimination that also was working to detect the luminance contrast thresholds or the interaction between a mechanism with low luminance contrast sensitivity and a mechanism with high chromatic discrimination. The observation of low luminance contrast sensitivity and the high chromatic discrimination within the mechanisms that underly the contrast discrimination (using our novel stimulus), suggests that P pathway (and probably K pathway) potentially has a fundamental role in these mechanisms. The P pathway could be the common trunk for new streams that encode exclusively colour information and that process colour and lumiance combined information in the visual cortex^22,23^. It is probable that other visual pathways also contribute to the inputs of the colour and luminance combined pathways^23^.

Using the novel stimulus, a set of chromaticities was used to introduce chromatic noise. The influence of the spectral content of the chromatic noise over the luminance discrimination was examined in Experiment 3. Five sets of chromaticity noise were used located in different places on the CIE1976 colour space: C1 (yellowish chromaticities), C2 (no colour predominance), C3 (bluish chromaticities), C4 (greenish chromaticities), and C5 (reddish chromaticities). These reference chromaticities were the same used in Regan *et al*. (1994) to estimate colour ellipses discrimination around them. Similar luminance discrimination was observed under these different chromatic adaptation states. This indicated that the mechanism was dependent on the masking effect of the chromatic noise over the luminance contrast discrimination. The chromatic content of the noise seemed to have no effect on this phenomenon.

All the results presented are comparative, as such the use of the “C” as the stimuli is constant across the different sessions. It is not clear the impact on the results if another stimulus was used. Several studies have discussed about misreading in the Ishihara test^55–58^. The normal trichromat observer can read incorrectly figures that looks like other figures, for example the numbers 3 and 8. The use of other symbols could have led to slightly changes in the task we did in the present study, but the real impact of their use in the threshold values needs for additional investigations.

Our results were consistent using two test platforms with different colour depth (MacBook + LCD, 10 bits per channel; ViSaGe system + CRT, 14 bits per channel). We found no systematic difference for results of the observers tested using both systems, and it enables the use of the new stimulus for applications programmed in low-cost system. We also observed that there was good replicability of the results and some learning effect after three consecutive sessions. For the higher values of the chromatic noise (0.06 and 0.03 u’v’ units), there was a significant decreasing of the contrast thresholds after the first session.

The novel stimulus that uses mosaic design and chromatic noise to mask luminance discrimination opens up an alternative way to investigate the colour and luminance interactions. These data, increase the comparability of the results obtained from colour discrimination using pseudoisochromatic stimulus and results of luminance contrast discrimination. New investigations with this novel stimulus are needed to further elucidate the mechanism that underlies the impairment of luminance discrimination in the presence of chromatic noise.

## Methods

The influence of the chromatic and spatial noises on the luminance discrimination, was investigated using three different experimental conditions: #1, #2, and #3. Experiment #1 was designed to determine the luminance contrast threshold as a function of the chromatic noise length; Experiment #2 aimed to compare the results estimated from mosaic and non-mosaic stimuli; and finally experiment #3 compared the estimated luminance contrast threshold using different chromatic noises. All subjects exhibited normal visual acuity or corrected at 20/20. Each participant had their colour vision evaluated by three assessments; Ishihara test, Cambridge Colour Test, and Color Assessment and Diagnosis test. Additionally, the subject’s general health history was also noted; no subject reported to suffer of diabetes, high blood pressure, neurological diseases, exposure to organic solvents or any other disease that could impair the visual function. All participants gave written informed consent for both study participation, and publication of identifying information/images. The Ethics Committee on Human Research of the Tropical Medicine Nucleus of the Federal University of Pará approved all procedures reported in the present study (report ##570.434). All methods were performed in accordance with Declaration of Helsinki.

### Experiment #1

**Figure 6:**
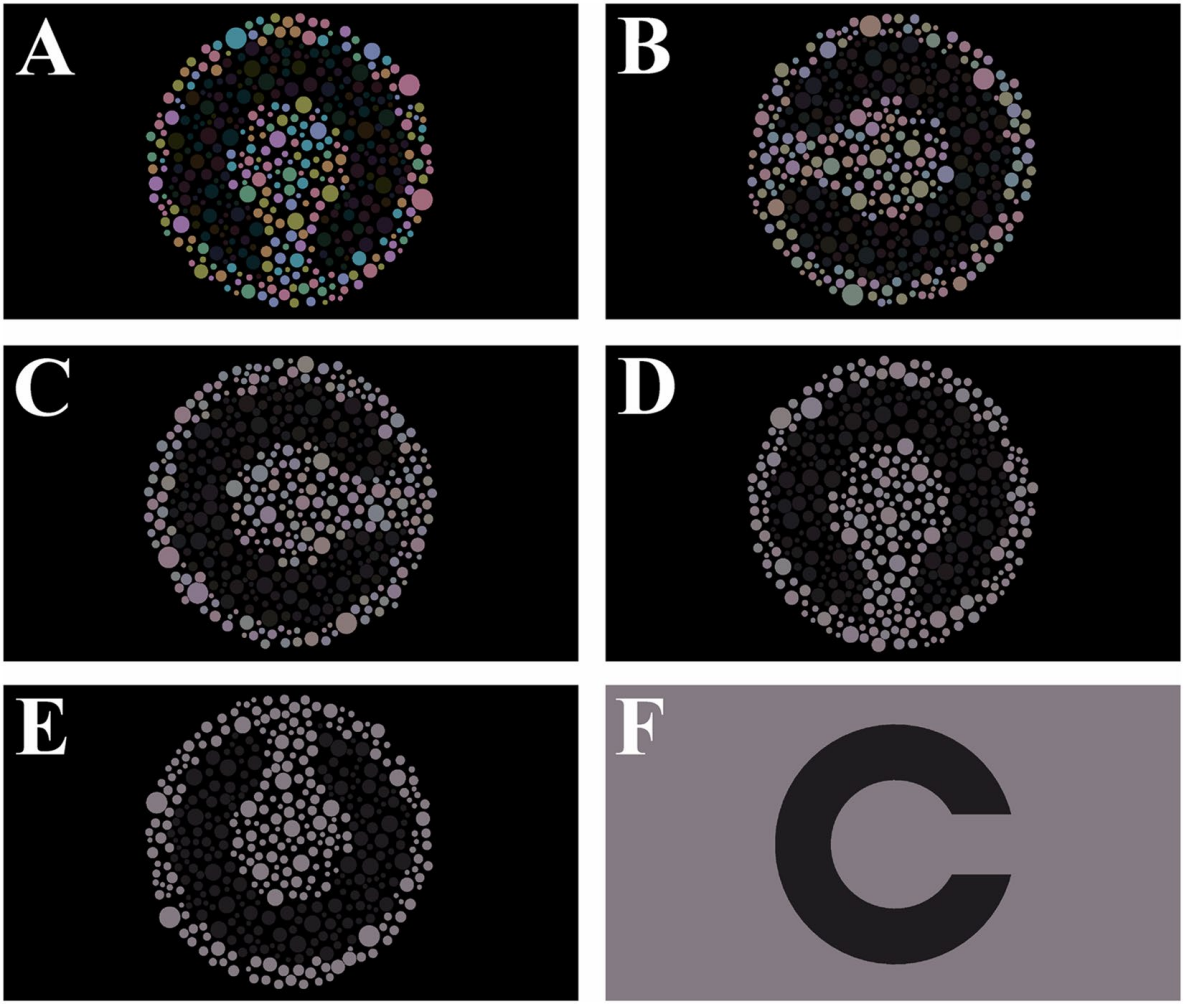
Stimuli used in Experiments #1 and #2. (**A**–**E**) The mosaic stimulus had a size and chromatic noise with different vector lengths: (**A**) 0.06; (**B**) 0.03; (**C**) 0.015; (**D**) 0.0075; (**E**) no chromatic noise. In Experiment #1, the influence of the chromatic noise length on the luminance discrimination was investigated. The contrast luminance thresholds estimated from the mosaics were compared to the one estimated from the non-mosaic stimulus (**F**) in Experiment #2.

**Figure 7:**
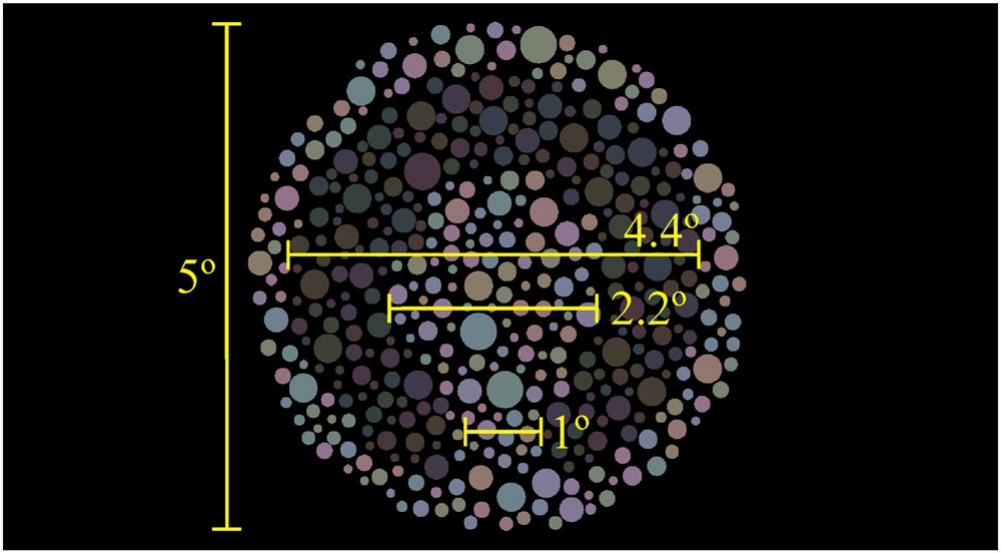
Spatial dimensions of the stimulus. The total diameter of the mosaic was 5° of visual angle; the outer and inner diameters of the Landolt-C was 4.4° and 2.2° of visual angle, respectively; and the Landolt-C gap was 1° of visual angle.

Forty normal trichromat subjects (mean age of 27.3 ± 3.91 years old) participated in this portion of the investigation. The stimulus was presented within a software programmed in MATLAB language environment (MATLAB 2012b, Mathworks, Natick, MA, USA) on a MacBook PRO platform (Apple Inc., Palo Alto, USA) the liquid crystal display (LCD) had 1680 × 1050 pixels of spatial resolution. The 17” MacBook Pro drove a NVIDIA GeForce 8600 M GT graphics processor with 512 MB of GDDR3 SDRAM and dual-link DVI and 10 bits of colour resolution per channel. We used a chromameter (CS-100A, Konica Minolta, Osaka, Japan) to calibrate the display. All chromatic calculations of the present study assumed a two degrees observer angle and a D65 illuminant. Target and background were formed by a mosaic of about 428 circles. We used 16 different mosaics. The spatial dimension of the circles ranged between 0.1226 and 0.4876 degrees. We do not consider that the spatial dimensions of the mosaic elements could insert some cue in the luminance discrimination task. The chromatic noise was composed by 8 different chromaticities angled in 0, 45, 90, 135, 180, 225, 270 and 315 degrees from a reference chromaticity in the CIE1976 colour space (u = 0.219; v’ = 0.48). The chromatic noise was quantified by the vector length from the reference chromaticity to each one of the 8 chromaticities. We used vector lengths of 0.06, 0.03, 0.015, 0.0075 u’v’ units, and one condition without the chromatic noise, i.e. all the circles had the reference chromaticity. The Landolt C target was subgroup of circles which differed in luminance from the background. Figure 6A–E shows the stimuli in five chromatic noise conditions. The background covered 5°of visual angle, the outer and inner diameters of the target were 4.4° and 2.2° of visual angle, respectively, and the C gap had 1° of visual angle (Fig. 7). The psychophysical experiment consisted of estimating the luminance threshold using a four alternatives forced-choice procedure. The task to perform was to identify the orientation of the Landolt C gap (up, down, left, and right). An adaptive stochastic approximation staircase method controlled the luminance of the target, which started with 2 cd/m^2^. Throughout the duration of the experiment, the background luminance was kept at 40 cd/m^2^. The staircase rule was that after two correct responses the target luminance increased, and after one wrong response the target luminance decreased. The step size of target luminance increase/decrease followed the equation (1). The target luminance (targetlum) of the trial (t) was summed (after two correct responses) or decreased (after one wrong response) by the difference between the background luminance (bglum) and target luminance of the previous trial times a factor (f), which value was 0.5 for increase luminance step and 1.5 for decrease luminance step.

$${\rm{targetlum}}({\rm{t}})={10}^{\mathrm{log}10({\rm{targetlum}}({\rm{t}}-1))\pm [\mathrm{log}10({\rm{bglum}})-\mathrm{log}10({\rm{targetlum}})]\times {\rm{f}}}$$

**Figure 8:**
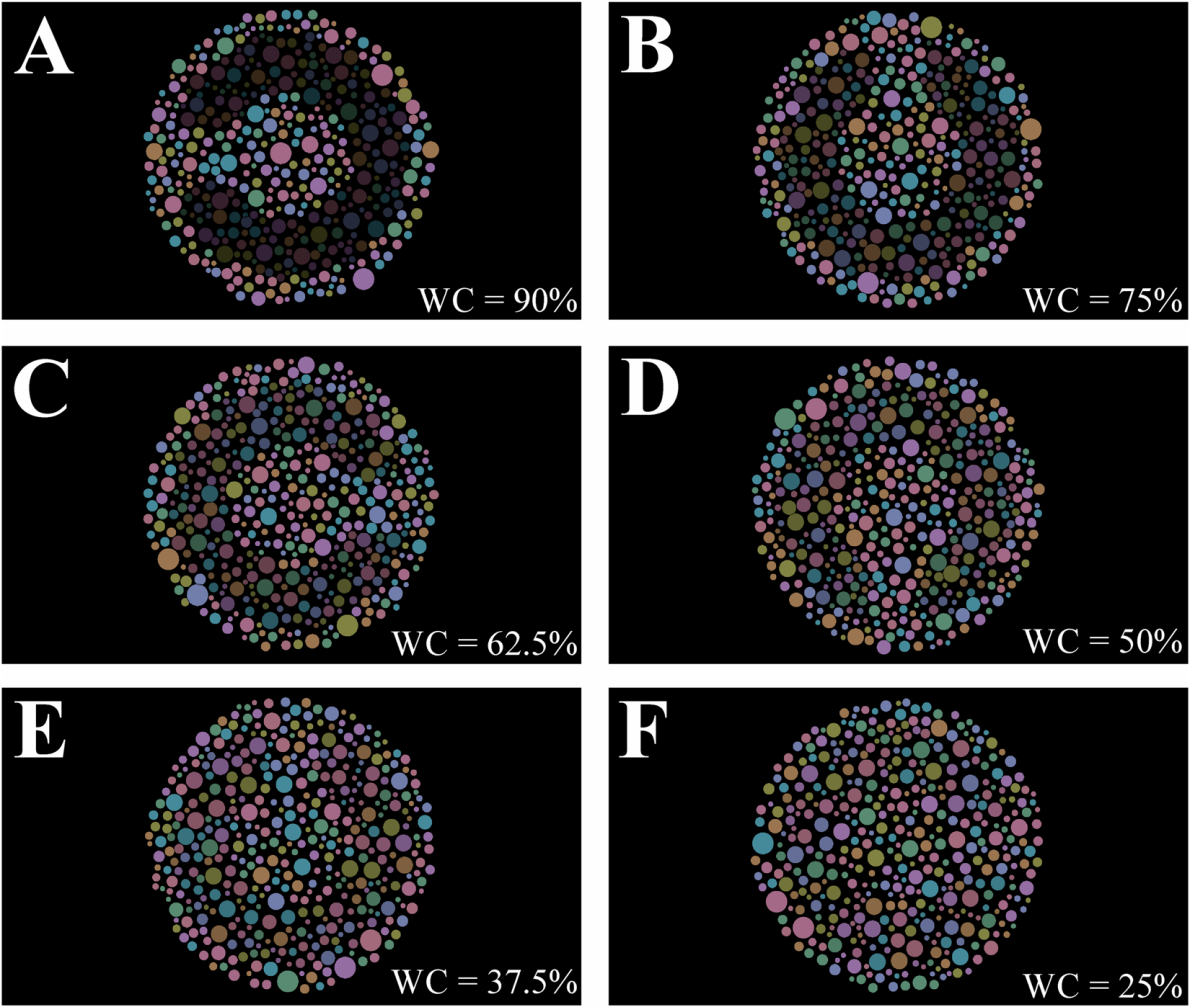
Examples of the stimuli with different Weber contrast between the target and the background. (**A**) Target: 4 cd/m^2^; Background = 40 cd/m^2^; Weber contrast = 90%. (**B**) Target: 10 cd/m^2^; Background = 40 cd/m^2^; Weber contrast = 75%. (**C**) Target: 15 cd/m^2^; Background = 40 cd/m^2^; Weber contrast = 62.5%. (**D**) Target: 20 cd/m^2^; Background = 40 cd/m^2^; Weber contrast = 50%. (**E**) Target: 25 cd/m^2^; Background = 40 cd/m^2^; Weber contrast = 37.5%. (**F**) Target: 30 cd/m^2^; Background = 40 cd/m^2^; Weber contrast = 25%.

After 12 reversals the staircase stopped, and the Weber contrast between the target and background was recorded. The contrast threshold was calculated averaging the last seven reversals. The forty participants performed the experimental procedure for twice. We also did the experimental procedure in a subgroup of thirteen participants for three consecutive trials to investigate the learning curve for the test. Figure 8 shows the stimulus in different luminance Weber contrast.

### Experiment #2

The Experiment #2 was the comparison between the Experiment #1 results with the luminance thresholds estimated using a non-mosaic stimulus. During the first session of Experiment 1, we also estimated the contrast threshold in a non-mosaic condition. The non-mosaic condition was randomly shown among the first session mosaic conditions. Using the same system, a non-mosaic stimulus was programmed (Fig. 6F). A Landolt-C target with the same dimensions as in Experiment 1 was centered on an isoluminant and isochromatic field. The psychophysical method to estimate the luminance threshold was the same as used in Experiment 1. We calculated the log_10_ of the ratio between the thresholds obtained with mosaics and the threshold obtained using non-mosaic stimulus in order to estimate their difference.

### Experiment #3

**Figure 9:**
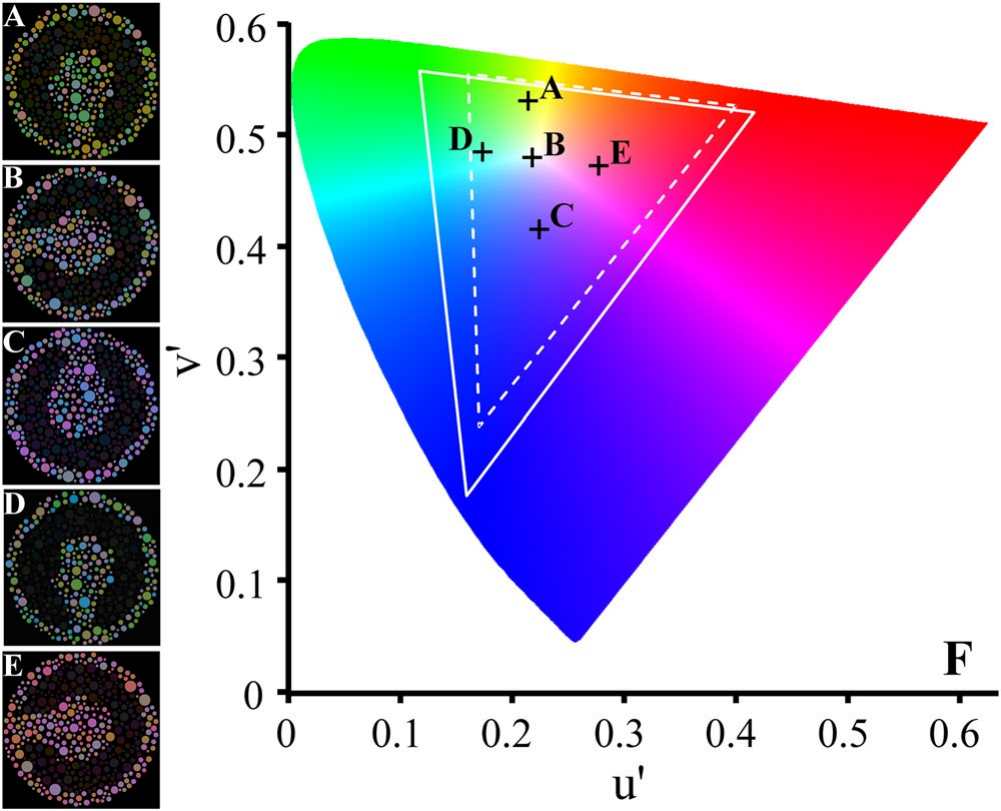
Stimuli used in Experiment #3. The chromatic noise was generated from five different reference chromaticities (**A**, C1; **B**, C2; **C**, C3; **D**, C4; C5, **E**). (**F**) CIE1976 color space showing the reference chromaticities (crosses) used to generate the chromatic noise. (**A**,**B**,**C**,**D** and **E**) represent the reference chromaticities of the chromatic noise described in the Methods section named as C1, C2, C3, C4, and C5, respectively. White line represents the CRT gamut and the dashed white line represents the LCD gamut. In Experiment #3, the influence of the different state of chromatic adaptation on the luminance contrast thresholds estimates was investigated.

Seven normal trichromats participated in this procedure (#3). We used ViSaGe system (Cambridge Research System, CRS, Rochester, UK) to present the stimulus on a CRT monitor with high spatial and temporal resolution (1680 × 1050 pixels, 75 Hz, 14 bits of colour resolution per channel). CRS toolbox for MATLAB was used to program the stimulus in MATLAB language environment (MATLAB 2012b, Mathworks, Natick, MA, USA) and to drive the ViSaGe graphic card (CRS). We used the same design of the mosaic stimulus described in Experiment #1, but the chromatic noise was modulated around by five reference chromaticities: C1: u’ = 0.215, v’ = 0.531; C2: u’ = 0.219, v’ = 0.48; C3: u’ = 0.225, v’ = 0.415; C4: u’ = 0.174, v’ = 0.485; C5: u’ = 0.278, v’ = 0.472 (Fig. 9). The same chromatic noise lengths used in experimental procedure #1 were also used in the third protocol (Experiment #3). The luminance contrast threshold was estimated similarly to that of Experiment #1.

### Data analysis

One-way ANOVA followed by a Tukey posthoc test was used to compare the contrast thresholds estimated in the different chromatic noise length in the Experiments #1 and #2. Two-Way ANOVA followed by a Bonferroni posthoc test was used to determine contrast thresholds estimated for the five chromatic noise lengths and three stimuli conditions of the Experiment #3. We considered the significance level of 5%.

## Limitations

The sample size of the Experiment #3 is different of the sample sizes of the Experiments #1 and #2.

## Conclusion

The present study introduces a new method to investigate luminance vision for basic science and clinical applications. A combination of spatial and chromatic noise impaired the luminance contrast threshold detection. The presence of one or more colour- and luminance-sensitive visual pathways could be physiological substrate of the mechanism that underlies the luminance contrast perception of this novel stimulus.

## References

[1] DiCarlo JJ, Zoccolan D, Rust NC (2012). How does the brain solve visual object recognition?. Neuron..

[2] Párraga CA, Brelstaff G, Troscianko T (1998). Color and luminance information in natural scenes. J. Opt. Soc. Am. A..

[3] Silveira LCL (2008). Division of labor between M and P visual pathways: different visual pathways minimize joint entropy differently. Psychol. Neurosci..

[4] Lee BB, Sun H (2011). Contrast sensitivity and retinal ganglion cell responses in the primate. Psychol. Neurosci..

[5] Lee BB, Sun H, Valberg A (2011). Segregation of chromatic and luminance signals using a novel grating stimulus. J Physiol..

[6] Lee BB (2011). Visual pathways and psychophysical channels in the primate. J Physiol..

[7] Dreher B, Fukada Y, Rodieck RW (1976). Identification, classification and anatomical segregation of cells with X-like and Y-like properties in the lateral geniculate nucleus of old-world primates. J. Physiol..

[8] de Monasterio FM (1978). Properties of concentrically organized X and Y ganglion cells of macaque retina. J Neurophysiol..

[9] de Monasterio FM (1978). Center and surround mechanisms of opponent-color X and Y ganglion cells of retina of macaques. J Neurophysiol..

[10] Creutzfeldt OD, Lee BB, Elepfandt A (1979). A quantitative study of chromatic organization and receptive fields of cells in the lateral geniculate body of the rhesus monkey. Exp Brain Res..

[11] Perry VH, Oehler R, Cowey A (1984). Retinal ganglion cells that project to the dorsal lateral geniculate nucleus in the macaque monkey. Neuroscience..

[12] Lee BB, Martin PR, Valberg A (1989). Amplitude and phase of responses of macaque retinal ganglion cells to flickering stimuli. J Physiol..

[13] Lee BB, Martin PR, Valberg A (1989). Sensitivity of macaque retinal ganglion cells to chromatic and luminance flicker. J Physiol..

[14] Lee BB, Martin PR, Valberg A (1989). Nonlinear summation of M- and L-cone inputs to phasic retinal ganglion cells of the macaque. J Neurosci..

[15] Dacey DM, Lee BB (1994). The “blue-on” opponent pathway in primate retina originates from a distinct bistratified ganglion cell type. Nature..

[16] Dacey DM (2000). Parallel pathways for spectral coding in primate retina. Annu. Rev. Neurosci..

[17] Hendry SH, Reid RC (2000). The koniocellular pathway in primate vision. Annu Rev Neurosci..

[18] Nassi JJ, Callaway EM (2009). Parallel processing strategies of the primate visual system. Nat Rev Neurosci..

[19] Hubel DH, Wiesel TN (1968). Receptive fields and functional architecture of monkey striate cortex. J Physiol..

[20] Chatterjee S, Callaway EM (2003). Parallel colour-opponent pathways to primary visual cortex. Nature..

[21] Shapley R, Hawken MJ (2011). Color in the cortex: single- and double-opponent cells. Vision Res..

[22] Johnson EN, Hawken MJ, Shapley R (2001). The spatial transformation of color in the primary visual cortex of the macaque monkey. Nat Neurosci..

[23] Li X (2015). Mixing of Chromatic and Luminance Retinal Signals in Primate Area V1. Cereb Cortex..

[24] Xing D, Ouni A, Chen S, Sahmoud H, Gordon J, Shapley R (2015). Brightness–Color Interactions in Human Early Visual Cortex. J. Neurosci..

[25] Mullen KT (1985). The contrast sensitivity of human colour vision to red-green and blue-yellow chromatic gratings. J Physiol..

[26] Switkes E, Bradley A, De Valois KK (1988). Contrast dependence and mechanisms of masking interactions among chromatic and luminance gratings. J. Opt. Soc. Am. A..

[27] Cooper B, Sun H, Lee BB (2012). Psychophysical and physiological responses to gratings with luminance and chromatic components of different spatial frequencies. J. Opt. Soc. Am. A..

[28] Souza GS (2014). Low number of luminance levels in the luminance noise increases color discrimination thresholds estimated with pseudoisochromatic stimuli. Front Psychol..

[29] Cormenzana Méndez I (2016). Color discrimination is affected by modulation of luminance noise in pseudoisochromatic stimuli. Front Psychol..

[30] Mollon, J. D. The origins of modern color science in *The Science of Color* (ed. Shevell, S. K.) 1–39 (Optical Society of America, 2003).

[31] Regan BC, Reffin JP, Mollon JD (1994). Luminance noise and the rapid determination of discrimination ellipses in colour deficiency. Vision Res..

[32] Mollon JD, Reffin JP (1989). A computer-controlled colour vision test that combines the principles of Chibret and Stilling. J Physiol..

[33] Mancuso K, Neitz M, Neitz J (2006). An adaptation of the Cambridge Colour Test for use with animals. Vis. Neurosci..

[34] Mancuso K (2009). Gene therapy for red-green colour blindness in adult primates. Nature..

[35] Paramei GV (2012). Color discrimination across four life decades assessed by the Cambridge Colour Test. J. Opt. Soc. Am. A..

[36] Goulart PR (2008). A computer-controlled color vision test for children based on the Cambridge Colour Test. Vis Neurosci..

[37] Barbur, J., Rodriguez-Carmona, M., Evans, S. & Milburn, N. Minimum color vision requirements for professional flight crew, part 3: recommendations for new color vision standards. CAA Paper 2009/04 (2009).

[38] Ripamonti, C., Kalwarosky, S. & Nardini, M. A novel colour discrimination test suitable for low vision observers. In: 12^th^ International AIC Congress, 2014.

[39] Gur M, Akri V (1992). Isoluminant stimuli may not expose the full contribution of color to visual functioning: spatial contrast sensitivity measurements indicate interaction between color and luminance processing. Vision Res..

[40] Mullen KT, Losada MA (1994). Evidence for separate pathways for color and luminance detection mechanisms. J Opt Soc Am A..

[41] Chaparro A, Stromeyer CF, Kronauer RE, Eskew RT (1994). Separable red-green and luminance detectors for small flashes. Vision Res..

[42] Stromeyer CF, Kronauer RE, Ryu A, Chaparro A, Eskew RT (1995). Contributions of human long-wave and middle-wave cones to motion detection. The Journal of Physiology.

[43] Mullen KT, Cropper SJ, Losada MA (1997). Absence of linear subthreshold summation between red-green and luminance mechanisms over a wide range of spatio-temporal conditions. Vision Res..

[44] Conway BR (2014). Color signals through dorsal and ventral visual pathways. Vis Neurosci..

[45] McAnany JJ, Alexander KR (2008). Spatial frequencies used in Landolt C orientation judgments: relation to inferred magnocellular and parvocellular pathways. Vision Res..

[46] Gillespie-Gallery H, Konstantakopoulou E, Harlow JA, Barbur JL (2013). Capturing age-related changes in functional contrast sensitivity with decreasing light levels in monocular and binocular vision. Invest Ophthalmol Vis Sci..

[47] Watanabe A, Pokorny J, Smith VC (1998). Red-green chromatic discrimination with variegated and homogeneous stimuli. Vision Res..

[48] Kaplan E, Shapley RM (1986). The primate retina contains two types of ganglion cells, with high and low contrast sensitivity. Proc. Natl. Acad. Sci. USA.

[49] Purpura K, Kaplan E, Shapley RM (1988). Background light and the contrast gain of primate P and M retinal ganglion cells. Proc. Natl. Acad. Sci. USA.

[50] Kaplan E, Shapley RM (1982). X and Y cells in the lateral geniculate nucleus of macaque monkeys. J Physiol..

[51] Hicks TP, Lee BB, Vidyasagar TR (1983). The responses of cells in macaque lateral geniculate nucleus to sinusoidal gratings. J Physiol..

[52] Edwards DP, Purpura KP, Kaplan E (1995). Contrast sensitivity and spatial frequency response of primate cortical neurons in and around the cytochrome oxidase blobs. Vision Res..

[53] Pokorny J, Smith VS (1997). Psychophysical signatures associated with magnocellular and parvocellular pathways contrast gain. J Opt Soc Am A..

[54] Linhares JM (2016). Assessing the effects of dynamic luminance contrast noise masking on colour discrimination test. J Opt Soc Am A..

[55] Birch J, McKeever LM (1993). Survey of the accuracy of new pseudoisochromatic plates. Ophthalmic Physiol Opt.

[56] Birch J (1997). Efficiency of the Ishihara test for identifying red-green colour deficiency. Ophthalmic Physiol Opt.

[57] Rodriguez-Carmona M, O’Neill-Biba M, Barbur JL (2012). Assessing the severity of color vision loss with implications for aviation and other occupational environments. Aviat Space Environ Med.

[58] Sloan LL, Habel A (1956). Tests for color deficiency based the pseudoisochromatic principle: a comparative study of several new tests. AMA Arch Ophthalmol.

